# Call for One Health at the World Health Assembly

**DOI:** 10.1016/j.eclinm.2022.101490

**Published:** 2022-05-25

**Authors:** eClinicalMedicine

The 75th World Health Assembly (WHA) will be held in May, 2022 in Geneva. As the decision-making body of the WHO, four key pillars will guide the meeting: (1) universal health coverage; (2) health emergencies; (3) better health and wellbeing; and (4) WHO's provision of support. The pillars will form the backbone for discussion at the summit and development of resolutions to improve health outcomes worldwide.

The WHA will be a timely occasion to discuss and push once more for the One Health approach. Following the outbreak of a global pandemic, never before has it been so important to recognise the interconnectedness of human life, veterinary health, and planetary health. The need for closer collaboration must be incorporated at the WHA to contend with the extent of our health challenges. Action is needed especially around vector-borne disease, food security, and climate-related health emergencies. Progress at this year's WHA will depend on the acknowledgment of the interconnectedness of human health with our environment and resolutions reasoned through the foundations of One Health.

Vector-borne diseases epitomise the human–animal interaction in the environment. These include many of the world's deadliest illnesses, such as dengue, yellow fever, zika, and malaria, which are transmitted by mosquitoes or ticks. Such diseases cause more than 700 000 deaths annually, hitting the lowest-income countries hardest. The health risks of vector-borne diseases are increasing with globalisation and climate change, both of which enable pathogens to spread and mutate faster. A milder climate and deforestation are expected to widen the distribution of disease vectors such as mosquitoes and ticks, and increase their numbers, while prolonging seasonal activity. Deforestation can disrupt ecosystems in ways that favour larval development and increase adult survivorship of mosquitoes. Although resolutions revolving around human health alone can be of use, such resolutions cannot make headway in isolation. Reducing our carbon footprint, while protecting forests will give the measures taken by WHO to tackle these diseases a fighting chance. Vector-borne disease features heavily in the WHA agenda. Outcomes from the WHA should enforce health agencies to work in collaboration with environmental legislators, promoting interactional benefits.

Recognising the One Health approach, the increasing effect of climate change on our world's health cannot be ignored at the WHA. Aside from the more visible effects of climatic changes in terms of floods, heat waves, food and water shortages, and forced migration, the impact on vector-borne diseases is increasingly concerning. Some disease areas show the reliance of the One Health foundations upon one another, whereas others exemplify how protection of one of the foundations will lead to synergistic benefits. The burden of conditions such as cardiovascular disease and obesity could be reduced by dietary solutions envisioned through the One Health lens.

A review published in the eClinicalMedicine by Giulia I Wegner and colleagues explores how the increasing incidence of infectious disease outbreaks is linked to deforestation for food production. Demand for meat products has led to intensive farming practices, in closer proximity to where people live, increasing the likelihood of pathogens jumping between species. Intensive farming is also associated with the overuse of antimicrobial drugs, encouraging antibiotic resistance. Intensively farmed livestock have reduced genetic diversity, making the herds more susceptible to pathogens. All these practices have tremendous effects on animal health and, acknowledging the One Health approach, also on human life. To sustain a healthy planet, diets need to adapt, and this adaptation will improve human health synergistically.

The programme for this year's WHA suggests ambitious aims and WHO is a powerful body, but we need more actionable goals and practice: without the coordination of environmental protection, and a substantial rethinking of farming practices and human diets, preparedness measures and prevention strategies are futile. 50 years since the establishment of the UN Environment Programme, global health discourse has continued to neglect the relationships between animals, ecosystems, and human health. Environmental protection bolsters health for all, through both sustainable food practices and resilient ecosystems in which wild animals live. The COVID-19 pandemic illuminated the legitimacy of the One Health concept in preventing epidemics. WHO is in support of a One Health approach, yet this support has not been effectively incorporated into actions thus far. Without cross-sector collaboration between WHO and environment ministers to avert the climate crisis with environmental legislation imposed, progress is difficult. We once more call for the incorporation of the One Health mindset into all health decisions, especially at the WHA, but also across all health governance at the local, national, and international levels. One Health can turn the tide.

